# Progress and future of molecular pain

**DOI:** 10.1186/1744-8069-3-2

**Published:** 2007-01-10

**Authors:** Jianguo Gu, Min Zhuo

**Affiliations:** 1 Department of Oral & Maxillofacial Surgery, McKnight Brain Institute and College of Dentistry, University of Florida, Gainesville, Florida 32610, USA; 2 Department of Physiology, Faculty of Medicine, University of Toronto, Toronto, Ontario M5S 1A8, Canada

## Abstract

Since its launch at the beginning of 2005, *Molecular Pain *has published pain research articles that cover broad areas including: genetics, molecular and cellular biology, synaptic and neuronal mechanisms, novel animal models and human functional imaging studies. One important feature of *Molecular Pain *is its high speed in manuscript processing and publication, making the journal one of the best places for pain researchers to publish their novel findings.

## Editorial

The new year of 2007 marks the two-year anniversary of *Molecular Pain*. We take this opportunity to wish our authors and readers a productive and successful new year, and we hope you will be with Molecular Pain in the coming years. We thank reviewers and editorial board members of Molecular Pain for your strong support. Your contribution, support, and suggestion will remain to be important for Molecular Pain in the coming years. Molecular pain research field is expanding and publication with our journal will become more active.

Two years ago, in the preparation for launching *Molecular Pain*, some pain researchers questioned whether we were indeed in the era of "molecular pain" and whether *Molecular Pain *was an appropriate title for this journal. Instead of waiting for a complete answer, *Molecular Pain *editors went ahead set up the platform for our pain researchers who share the same vision with us. Over the past two years, *Molecular Pain *has published papers that covered pain topics from genetics, molecular biology, synaptic mechanism, to novel animal models and human functional imaging studies. *Molecular Pain *is now recognized by many pain researchers and neuroscientists as an effective forum for communicating their novel research findings. The effectiveness comes from our online publication in a timely fashion and the open access to everyone around the world without any barrage.

**Figure 1:**
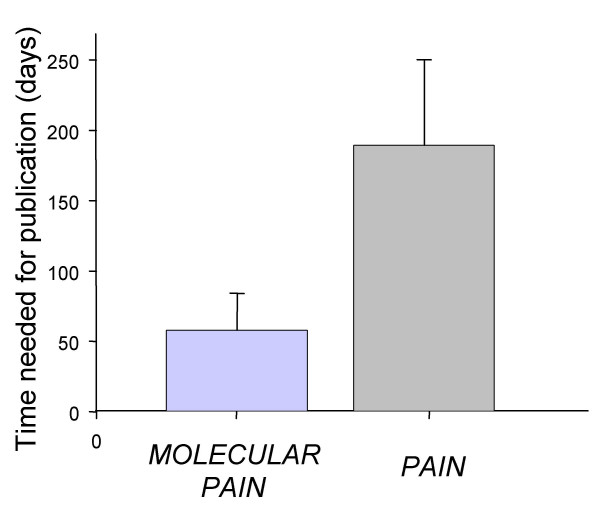
**Days from the submission to the publication in *Molecular Pain *or in *Pain***. The comparison is based on twenty articles randomly selected from each journal in 2006.

The publication punctuality is essential for researchers who have novel findings in today's highly competitive "research market". *Molecular Pain *reviewers, editors and *BMC *have been working hard and making every effort to ensure timely processing and publishing papers that are submitted to *Molecular Pain*. Powered by *BMC *online system for manuscript processing, publication speed of *Molecular Pain *is ranked on the top list among peer-review scientific journals. In average, it only takes 60 days from receiving a manuscript to its publication online in *Molecular Pain*. On the other hand, professional journals such as *Pain *will take about 180 days to publish a manuscript, about 3 times longer than *Molecular Pain *(see Figure 1). We will continue to let authors enjoy rapid publication with *Molecular Pain *in 2007 and further reduce processing time for papers with high novelty.

Submission to *Molecular Pain *is growing over the past two years. In the year 2005, about 40% of papers were solicited submission. However, in 2006, over 90% of papers were unsolicited submission. Submission now comes from both well-known pain research groups as well as from new investigators from different countries and regions. We are pleased to see multiple submissions from same research groups, which show their trust and satisfaction to *Molecular Pain*.

Many articles published in *Molecular Pain *have been "hit" for over thousands of times by readers over a relatively short time period following their publication. This indicates that articles published in *Molecular Pain *gain high access and readership, promising a high impact in the near future. Indeed, a number of articles published in *Molecular Pain *have been highly cited in literature in less than two years of their publication. The rate of citation for these articles published in *Molecular Pain *is comparable to papers published in well established journal such as *Journal of Neuroscience*. This clearly shows the advantage of open access and online publication with *Molecular Pain*. *Molecular Pain *has been continuously tracked by *PubMed *and *Medline *since its launch at the beginning of 2005. Because of the steady growth and good quality of papers published in *Molecular Pain*, *ISI *has recently approved for tracking *Molecular Pain *publications for official impact factor, which will be announced as a public record in two years.

Over the past two years we have received a number of requests from pain researchers and neuroscientists around the world who are interested in joining the *Molecular Pain *editorial board. We have expanded the editorial board to strengthen the research area including ion channels and brain imaging. We once again thank those who are voluntarily coming up to serve as editorial board member to strengthen *Molecular Pain*. Several other areas such as human genetics, clinical pain research, and drug discoveries remain to be strengthened in the *Molecular Pain *editorial board. We will welcome pain researchers with expertise in these areas to join the editorial board. Please contact us if you are interested in joining us to expand molecular pain research field.

In addition to our main focus on publication, *Molecular Pain *has sponsored "The First International Conference of Memory, Synapses, and Pain" held in Toronto in the August of 2006. The meeting abstract has been published online in *Molecular Pain*. *Molecular Pain *will continue to sponsor this meeting, which will be held again in the fall of 2007 in Toronto. Publication of meeting abstracts with *Molecular Pain *provides a useful record. *Molecular Pain *will provide this platform for other pain-related meetings, and we welcome meeting organizers to contact *Molecular Pain *editors for an arrangement to publish meeting abstract and other related information.

Over the past two years, contributors to *Molecular Pain *have enjoyed low introductory publication charges offered by our publisher BMC. BMC has recently requested to adjust the publication charge comparable to other online journals. After our negotiation with BMC, the change of publication charges will be modest and we will continue to enjoy a relatively low publication cost compared with other online journals in BMC.

In conclusion, *Molecular Pain *has achieved its primary goal in the first two years. We will continue to provide the best service to our authors and readers, making *Molecular Pain *a high-impact scientific journal.

